# (iv) Enhancing the safety and reliability of joint replacement implants

**DOI:** 10.1016/j.mporth.2012.05.006

**Published:** 2012-08

**Authors:** Louise M. Jennings, Mazen Al-Hajjar, Claire L. Brockett, Sophie Williams, Joanne L. Tipper, Eileen Ingham, John Fisher

**Affiliations:** **Louise M Jennings MEng PhD** Principal Research and Innovation Fellow, Institute of Medical and Biological Engineering iMBE, University of Leeds, Leeds, UK and Leeds Musculoskeletal Biomedical Research Unit, Leeds Teaching Hospital Trust, Leeds, UK. Conflict of interest: none; **Mazen Al-Hajjar BEng** Research Engineer, Institute of Medical and Biological Engineering iMBE, University of Leeds, Leeds, UK and Leeds Musculoskeletal Biomedical Research Unit, Leeds Teaching Hospital Trust, Leeds, UK. Conflict of interest: none; **Claire L Brockett MEng PhD** Senior Research Fellow, Institute of Medical and Biological Engineering iMBE, University of Leeds, Leeds, UK and Leeds Musculoskeletal Biomedical Research Unit, Leeds Teaching Hospital Trust, Leeds, UK. Conflict of interest: none; **Sophie Williams BSc PhD** Senior Lecturer, Institute of Medical and Biological Engineering iMBE, University of Leeds, Leeds, UK and Leeds Musculoskeletal Biomedical Research Unit, Leeds Teaching Hospital Trust, Leeds, UK. Conflict of interest: none; **Joanne L Tipper BSc PhD** Senior Lecturer, Institute of Medical and Biological Engineering iMBE, University of Leeds, Leeds, UK and Leeds Musculoskeletal Biomedical Research Unit, Leeds Teaching Hospital Trust, Leeds, UK. Conflict of interest: none; **Eileen Ingham BSc PhD** Professor, Institute of Medical and Biological Engineering iMBE, University of Leeds, Leeds, UK and Leeds Musculoskeletal Biomedical Research Unit, Leeds Teaching Hospital Trust, Leeds, UK. Conflict of interest: none; **John Fisher CBE BSc PhD DEng** Professor, Institute of Medical and Biological Engineering iMBE, University of Leeds, Leeds, UK and Leeds Musculoskeletal Biomedical Research Unit, Leeds Teaching Hospital Trust, Leeds, UK. Conflict of interest: none

**Keywords:** hip replacement, knee replacement, pre-clinical simulation, stratified approach for enhanced reliability SAFER, wear

## Abstract

A new **S**tratified **A**pproach **F**or **E**nhanced **R**eliability (**SAFER**) pre-clinical simulation testing of joint prostheses is presented in this article. The aim of this approach is preclinical systematic testing of wear performance in the much wider envelope of conditions found clinically rather than relying only on the standard testing conditions that are currently used. The approach includes variations in surgical delivery, variations in kinematics, variations in the patient population and degradation of the biomaterial properties. Clinical experience of existing prostheses has been used to validate the new *in vitro* methods.

## Introduction

Hip and knee joint replacements have been used extensively over the past 50 years generally with a high level of success. However, the current generation of patients is placing higher demands on prostheses, and they are being increasingly used in younger and more active patients. Patients now have higher expectations of their prostheses, encompassing reliability, function and lifetime duration with ‘50 active years after 50^®^’. Pre-clinical tribological simulation can be used to determine the performance of a prosthesis under a set of physiological kinematic conditions expressed as friction, wear, wear debris and biological activity.^1–3^ However, this testing has predominantly been performed under a set of idealized standard gait conditions; for the standard patient in which the prosthesis has been implanted with perfect surgical technique and under a single activity. Since both patients and surgeons now have higher expectations of joint replacements, there is a requirement for pre-clinical testing to be enhanced, beyond the current standards and levels of compliance in order to cover a much wider set of clinically relevant conditions, to demonstrate the prosthesis is robust to these non-perfect conditions.

Until the 1990's there was very limited pre-clinical testing of hip and knee joint replacements. The development of standard pre-clinical tribological simulation methods for hip joints was driven by clinical failures due to polyethylene wear debris induced osteolysis.^1–3^ From the 1990s up until the present time, standard pre-clinical tribological tests^4–9^ have been used to assess performance by industry and regulatory bodies. ISO and ASTM standards were developed, which detail a standard walking cycle test. These standards reflect a prosthesis with no degradation, a single activity (walking), a standard patient of average weight, and accurate surgery and alignment of the prosthesis. Such tests are often used to show equivalence to designs already in clinical use. For conventional polyethylene in the hip, the average wear rate under such standard conditions matched the average clinical wear rate.^10^ However, there was much greater variation in clinical wear rates.^1–3,10,11^ Pre-clinical testing has been subsequently used to demonstrate improvements in material wear properties, such as for cross-linked polyethylene, which showed a 5 fold reduction in wear compared to conventional polyethylene.^12^ For alternative hard-on-hard bearings, clinically these have shown a highly skewed distribution with outliers, and this has not been replicated in the laboratory under standard conditions.^13,14^ In the knee, there has been little clinical evaluation of tibial wear rate, but modes of damage and the location of the wear area can vary significantly between retrieved samples.^15^

An overall comparison of *in vitro* wear under standard conditions and *in vivo* wear reveals that the standard walking cycle simulation *in vitro* may replicate average *in vivo* wear rates but it does not replicate the variation and spread of *in vivo* wear rates, or the outliers, which may be up to 100 fold greater than the average. The standard walking cycle simulation has been effective in developing and evaluating designs that have a reduced average wear rate, such as cross-linked polyethylene, but factors that determine the level of (low) failure rates cannot be evaluated through standard walking cycle simulator studies and determination of average wear rates. To understand, evaluate and reduce failure rates further, we need to utilize non-standard or adverse conditions in *in vitro* simulations.

This article presents a stratified approach to pre-clinical testing, which has been developed over the past 10 years, aimed at enhancing the safety and reliability of total joint replacements.

## Methods and the stratified approach

The current standard for pre-clinical tribological evaluation of joint prostheses considers the following:

• A prosthesis that does not degrade
• A correctly positioned prosthesis (correct rotational and translational positions)
• A standard walking cycle
• An average standard patient, anatomy and physiology.

These standard conditions have been used to generate an *in vitro* bank of data of some billion walking cycles, which has then been compared to average clinical wear rates.^4–14^

Using a stratified approach to pre-clinical testing a number of test methodologies have been developed to systematically address a wider envelope of conditions, which include the following:

• Effect of variations and changes in the prosthetic device, such as femoral head damage^6^ and oxidative degradation of polyethylene.^16^
• Variations in surgical delivery and component positioning. Two principal types of component mal-positioning can occur, rotational mal-positioning and translational mal-positioning.^17–27^ Rotational mal-positioning is associated with the acetabular cup inclination angle, with steeply inclined cups moving the tribological contact patch towards the rim of the cup, resulting in edge loading. In a well-positioned prosthesis, the centres of rotation of the femoral head and the acetabular cup are matched. Translational mal-positioning conditions occur when these centres of rotation are separated. If the level of separation exceeds the radial clearance of the bearing couple, edge loading may occur. Translational mal-positioning may occur due to several clinical factors such as head offset deficiency, medialized cup, stem subsidence, impingement and laxity of the soft tissues.
• Different types of patient activities, such as joint kinematics in the hip,^5,28^ levels of activities, levels of loading, jogging, stop–start motion, level of swing phase load.^29,30^ Different input kinematics^31,32^ and the influence of femoral condylar lift off in the knee.^33^
• Different types of patients or patient conditions, such as variations in the natural lubricant,^29,34^ variation in anatomy and physiology and disease state and in the future inflammatory response, metal ion sensitivity.

The methodologies developed have been compared and validated against clinical wear rates where possible.

## Results

### Results for standard condition wear simulation

The normal range for average wear rates under standard conditions from pre-clinical simulation studies of hip replacements derived from a single laboratory is detailed in Table 1 below:

Essentially the highest wear rates were obtained for the metal on polyethylene combinations, with a significantly lower wear rate for the highly cross-linked polyethylene compared to conventional polyethylene. Clinical studies have also borne out this reduction in wear.^35,38,39^ It has been shown for conventional polyethylene that a wear rate of 40 mm^3^ per million cycles exceeds the critical volume of 500 mm^3^ needed to cause osteolysis at 10 years in an active patient. This means the reduction in wear rate with cross-linked polyethylene indicates this risk is substantially reduced. Under standard conditions in a low or medium demand patient a wear rate of less than 10 mm^3^/million cycles was indicative of acceptable wear performance for cross-linked polyethylene.

The alternative bearing combinations showed a decreasing trend in wear rate from under 1 mm^3^/million cycle for metal-on-metal, to under 0.1 mm^3^/million cycle for ceramic-on-metal and ceramic-on-ceramic. Although the metal-on-metal combinations showed a similar low wear rate for all sizes, this is not reflected in clinical experience, with a wide range of wear rates and many substantially higher wear rates being observed in individual patients.^27^ Wear rates above 1 mm^3^/million cycles were consistent with metal ion levels above 10 ppm, which may produce adverse reactions clinically.^27^

The lowest wear rates were obtained with the ceramic-on-ceramic bearings, under 0.1 mm^3^/million cycle. Wear at this level is very difficult to measure *in vitro* as it is on the limits of sensitivity of the measurement methods. However, as for metal-on-metal bearings standard simulation conditions have not been shown to replicate the clinical experience, which has revealed characteristic stripe wear and higher wear rates in ceramic-on-ceramic bearings.^17,18^ Further, the clinical wear rates of the order of 1 mm^3^/million cycles for ceramic do not appear to produce adverse biological reactions.

It is also important to consider the biological effects of the wear debris generated in terms of the type of reaction, inflammation and osteolysis or toxicity and necrosis, as well as the severity of the response to volumetric doses.^2,3^

Wear rates for the knee (medium size) under standard simulation conditions are shown in Table 2. The wear rate for conventional polyethylene in the knee was less than in the hip, which reflects the lower incidence of osteolysis in the knee under normal conditions. As in the hip, the wear rate was reduced with cross-linked polyethylene material and a wear rate of less than 10 mm^3^/million cycles may be considered acceptable performance for polyethylene in the knee.

### Results for examples of stratified approach for wear simulation

**Effect of changes in the prosthetic device:** changes in the prosthetic device could relate to materials, or design. For example, oxidative degradation of polyethylene was a problem historically resulting in delamination and osteolytic failure of hip and knee implants.^40^ New stabilized and cross-linked polyethylene's addressed this concern.^41^ However, other degradation mechanisms may also be taking place *in vivo*, such as a deterioration in the roughness of the smooth metallic femoral head in the hip.^10^ This would be particularly relevant as the prosthesis enters the second decade of its life *in vivo*. This has been investigated *in vitro* through developing a protocol for generating discrete scratches on the femoral heads, which was based on measurements of scratches in clinically retrieved prostheses.^10^ This damage produced a three-fold increase in the polyethylene wear for a size 28 mm hip replacement under standard conditions compared to a smooth head (Table 3). This would substantially increase the risk of osteolysis.

**Different surgical delivery and component positioning:** Table 4 details example wear rates under conditions associated with variation in the position of the hip prosthesis, in particular variation in the rotational position of the cup with an increased inclination angle of 60°, variation in the translational position of the head and cup with a microseparation of the centres by 0.5 mm and a combination of both conditions.

The effect of surgical delivery and component positioning on wear was dependent on the type of bearing. The current generation of ceramic-on-ceramic (Delta) and ceramic-on-metal produced wear rates below 1 mm^3^/million cycles under translational mal-positioning, which could be considered as an acceptable level of wear. Also it has previously been shown that there is no effect of rotational mal-positioning on the wear of ceramic-on-ceramic bearings.^13,26^ However, the wear of the metal-on-metal combinations were all above 1 mm^3^/million cycles, for both rotational and translational mal-positioning. The largest increase was for the larger diameter sub hemispherical surface replacement under translational and rotational mal-position, whereas the smaller diameter bearing showed the least increase. Such pre-clinical test results under adverse conditions were able to differentiate the performance between different materials and design, which is less evident under standard conditions. The results for metal-on-metal bearings provide a potential explanation for variation in clinical performance and outcomes with different designs. Further evidence of the value of this type of pre-clinical testing has been demonstrated in the development of surface engineered bearings for hips, where simulation of mal-positioning and rim loading was able to demonstrate failure of surface coating prior to the device entering clinical trials.^42^

**Different types of patient activities:** Table 5 details the variations in wear rate in the knee associated with changes in kinematics inputs. In particular, the effect of higher kinematic inputs through increased anterior-posterior translation and also the introduction of abduction-adduction femoral condylar lift off and medial-lateral translation were studied. All data presented is for a medium sized fixed bearing type of total knee replacement.

For conventional polyethylene the wear rate increased with higher kinematic inputs, with a greater increase for the lift off conditions. This would result in a corresponding increase in the risk of wear debris induced osteolysis. The increase in wear rate for the cross-linked polyethylene with increased anterior-posterior translation was not as substantial, showing a potential advantage of the cross-linked material under higher kinematic conditions.

**Different types of patients or patient conditions:** here we consider the patient variable of lubrication and the effect of lubricant composition on the friction coefficient of different bearing combinations in the hip (Table 6).

The metal-on-metal bearings had significantly higher friction factors compared to the other bearing materials. Protein concentration was shown to have a marked effect upon the friction factors of all the different bearings. For all material combinations, except metal-on-metal, increasing the protein concentration resulted in an increase in friction factor. In the metal-on-metal bearings the increased concentration of proteins appeared to improve the lubrication within the bearing.^29^

## Discussion

Patient demographics and their demands are changing, with younger and more active patients. This is in conjunction with increased expectations, not only of patients but also of healthcare providers, clinicians and regulators, who typically demand a success rate of greater than 90% at ten years. Wear debris induced osteolysis and failure remains the major cause of failure of joint replacements.^1–3^ Pre-clinical testing can play a key role in assessing the performance of joint replacements and historically pre-clinical wear testing under a standard walking cycle simulation has been shown to compare well with clinical data for some designs and materials. However, testing under such conditions as those of the ISO standard walking cycle have not been able to predict wear related failure or wear mechanisms clinically, neither have they been able to consistently differentiate the performance of different designs.

In order to enhance the safety and reliability of joint replacements, pre-clinical testing should include a wider range of clinically relevant conditions that are investigated in a systematic and rigorous fashion. This stratified approach to simulation should include changes in the prosthetic device, different patients activities, different types of patients or patient conditions and different surgical delivery and component positioning. Some examples of these conditions have been presented in this article but more research is needed to define the effect of different design variables on wear under a wider set of adverse conditions and a wider range of daily activities. Such a stratified approach to simulation will inform future design by defining the performance envelope of device, identifying failure mechanisms, informing clinicians and industry of conditions where a device might not be suitable and enhancing future product development for both design and material selection.

The first part of this article reported results under standard baseline test conditions for various bearing materials for historical and current joint replacements from our existing data set of over 5 billion test cycles. When combined with clinical experience, these results have defined indicative levels of acceptable performance in terms of wear rates for different materials. The differing bio-reactivity and cellular response meant that there appears to be a lower clinical tolerance to metal compared to polyethylene.

The second part of this article has introduced the concept of a new stratified approach for enhanced reliability (SAFER) pre-clinical simulation testing of joint prostheses, with many examples of how different clinically relevant conditions affect the wear rates and performance of various prostheses, and most importantly shown how different types of prostheses respond differently to the different conditions. The results from these wider sets of conditions have been compared with those obtained from the baseline standard walking cycle testing.

In the polyethylene hip there remain concerns around deterioration of the metallic head causing elevated wear and potentially increasing the risk of osteolysis. The scratch resistant ceramic head may lessen this risk, particularly if coupled with cross-linked polyethylene, which has shown reduced wear under standard conditions.^43,44^

In the hip, edge loading can occur when the femoral head contacts the rim of acetabular cup. This has been shown to produce a characteristic stripe on the femoral head for hard-on-hard bearing materials; indeed stripe wear was first observed on ceramic-on-ceramic retrievals by Nevelos et al.^17^ This stripe wear was not seen *in vitro* under standard testing conditions but can be produced *in vitro* through rotational or translational mal-positioning depending on the bearing material and design. For example, in ceramic-on-ceramic bearing, edge loading due to rotational mal-position did not cause stripe wear and increased wear, whereas translational mal-position (microseparation conditions) resulted in increased wear, stripe wear mechanisms, and the generation of bimodal micron sized wear particles, as seen on retrievals.^17,19,20,45^ Under standard conditions, the current generation of ceramic-on-ceramic Delta material when compared to previous generations, showed no distinguishable difference. However under adverse translational mal-positioning conditions the improved Delta material showed a greater resistance to wear.^26^ This highlights an advantage of the stratified approach, which is the capability to differentiate between different devices. This can reflect improvements in material performance. The ability to compare wear rates with clinical experience and performance is a fundamental requirement.

The wear of metal-on-metal bearings has been shown to be sensitive to both rotational and translational mal-positioning as well as design (cup coverage) and head size.^27^ When compared to ceramic-on-ceramic the absolute level of wear is much higher, and when combined with the increased reactivity, indicates a greater potential for clinical damage.

Under standard walking conditions, knee prostheses generally have low wear. In-vivo fluoroscopic studies have demonstrated significant variability in knee kinematics between patients, particularly the incidence of femoral condylar lift off from the tibial bearing.^46^ *In vitro* increased kinematics, through both increased anterior posterior translation and condylar lift off, produced elevated wear. Moderately cross-linked polyethylene has been shown to reduce wear under both standard conditions and higher kinematic conditions. Given the significant variability in knee kinematics between patients it is important to begin to test the performance of current knee designs under a wider range of clinically relevant kinematic conditions.

There is a need to consider other patients activities such as stair climbing, squatting, chair-rise and descent for example. However these activities may only impact on less than ten percent of the tribological cycles, whereas a mal-positioned prosthesis leading to edge loading in the hip or instability in the knee leading to femoral condylar lift off can cause high kinematic demand and increased wear on every step.

## Conclusion

This paper summarizes studies undertaken to date using our SAFER pre-clinical simulation and is based on a data bank of several million cycles of test data for hip and knee. However these findings only record some of the variations in clinical conditions. It is also important to recognize that there may be synergistic interactions between adverse conditions, and therefore a need to consider, in the future, parametric studies to examine the effect of independent and combined conditions.

In summary, average wear rates in current hip and knee joint replacements have been successfully reduced under standard conditions to acceptable low levels for each material type. Now it is necessary to focus on producing acceptable tribological performance in the much wider envelope of conditions found clinically. The new stratified approach for enhanced reliability (**SAFER**) pre-clinical simulation testing of joint prostheses presented in this article provides a frame work in which to systematically address this future challenge.

**Key learning points:**

• To date pre-clinical wear simulation has been performed under a standard walking cycle considering a correctly positioned prosthesis in an average standard patient.
• Average wear rates in current hip and knee joint replacements have been successfully reduced under standard conditions to acceptable low levels for each material type.
• To understand, evaluate and reduce (low) failure rates, we need to perform pre-clinical testing under the much wider envelope of conditions found clinically.
• Proposed stratified approach for enhanced reliability ‘SAFER’ pre-clinical simulation testing of joint prostheses challenges the bearing design with a stratified matrix of adverse conditions.

## Statement of commercial interest

JF, SW, CLB are paid consultant to DePuy.

JF and the University of Leeds receive royalty income from DePuy.

JF and EI are share holders and paid advisers to Tissue Regenix Group plc.

Research funding has been received from the following companies in the last 5 years: DePuy, Mathys, JRI, Ceramtec, Eurocoatings, Neu Biomechanics, Biomet, Simulation Solutions, Smith and Nephew.

## Figures and Tables

**Table 1:** Wear rates (mm^3^/million cycles) for hip replacements under standard conditions

Type	Metal on polyethylene^4–6,12^		Metal-on-metal^14,22,36,37^			Ceramic-on-metal^36^	Ceramic-on-ceramic^13,19,26^	
Conventional	Cross-linked 7.5–10 MRad	Alumina	Delta
Head size (mm)	28	28–36	28	36	39–55	28 to 36	28	28
Wear rate (mm^3^/million cycles)	25 to 40	5 to 10	0.1 to 1	0.4 to 0.8	0.1 to 0.4	0.02 to 0.1	0.02 to 0.1	0.02 to 0.1

**Table 2:** Wear rates (mm^3^/million cycles) for knee replacements under standard conditions

Type of material	Conventional polyethylene^8,9,33^	Cross-linked polyethylene 5 MRad
Wear rate (mm^3^/million cycles)	6 to 12	3 to 6

**Table 3:** Effect of deterioration in femoral head roughness in the hip on wear rates

Material	Conventional polyethylene	
Condition	Smooth^4–6^	Scratched head^10^
Wear rate (mm^3^/million cycles)	25 to 40	100 to 140

**Table 4:** Effect of different surgical delivery and component positioning on wear in the hip

Type	Metal-on-metal^25,36^			Ceramic-on-metal^36^	Ceramic-on-ceramic^19,26,45^	
Alumina	Delta
Head size (mm)	28	39	39	28	28	28
Adverse condition	Translational mal-position	Rotational mal-position	Translational rotational mal-position	Translational mal-position	Translational mal-position	Translational mal-position
Wear rate (mm^3^/million cycles)	0.5 to 3	1 to 10	8 to 14	0.1 to 0.5	0.5 to 1.8	0.5 to 0.25

**Table 5:** Variation in kinematic conditions in the knee

Type of material	Conventional polyethylene^31,32^	Cross-linked polyethylene 5 MRad	Conventional polyethylene^33^
Condition	Increased anterior posterior translation	Increased anterior posterior translation	Lift off
Wear rate (mm^3^/million cycles)	9 to 15	4.5 to 8.5	12 to 19

**Table 6:** Friction factors for size 28 mm hip replacements^29^

Type of hip	Metal on polyethylene	Metal-on-metal	Ceramic-on-metal	Ceramic-on-ceramic
100% serum	0.07	0.10	0.07	0.05
25% serum	0.06	0.12	0.05	0.04
Water	0.05	0.17	0.02	0.02

## References

[1] Fisher J. (1991). Tribology of artificial joints. J Eng Med.

[2] Ingham E., Fisher J. (2000). Biological reactions to wear debris in total joint replacement. Proc Inst Mech Eng.

[3] Ingham E., Fisher J. (2005). The role of macrophages in osteolysis of total joint replacement. Biomaterials.

[4] Bigsby R.J.A., Hardaker C.S., Fisher J. (1997). Wear of ultra-high molecular weight polyethylene acetabular cups in a physiological hip joint simulator in the anatomical position using bovine serum as a lubricant. Proc Inst Mech Eng.

[5] Barbour P.S.M., Stone M.H., Fisher J. (1999). A hip joint simulator study using simplified loading and motion cycles generating physiological wear paths and rates. Proc Inst Mech Eng.

[6] Barbour P.S.M., Stone M.H., Fisher J. (2000). A hip joint simulator study using new and physiologically scratched femoral heads with ultra-high molecular weight polyethylene acetabular cups. Proc Inst Mech Eng.

[7] Fisher J., McEwen H.M.J., Barnett P.I. (2001). Total knee replacement – practical considerations (i) Wear of polyethylene in artificial knee joints. Curr Orthop.

[8] Barnett P.I., McEwen H.M.J., Auger D.D., Stone M.H., Ingham E., Fisher J. (2001). Comparison of wear in a total knee replacement under different kinematic conditions. J Mater Sci Mater Med.

[9] McEwen H.M.J., Fisher J., Goldsmith A.A.J., Auger D.D., Hardaker C., Stone M.H. (2001). Wear of fixed bearing and rotating platform mobile bearing knees subjected to high levels of internal and external tibial rotation. J Mater Sci Mater Med.

[10] Tipper J.L., Ingham E., Hailey J.L. (2000). Quantitative analysis of polyethylene wear debris, wear rate and head damage in retrieved Charnley hip prostheses. J Mater Sci Mater Med.

[11] Ingham E., Fisher J., Stone M.H. (2003). Wear of historical polyethylene in hip prostheses. Biomechanical success and a biological failure. Hip Int.

[12] Galvin A.L., Tipper J.L., Jennings L.M. (2007). Wear and biological activity of highly crosslinked polyethylene in the hip under low serum protein concentrations. Proc Inst Mech Eng.

[13] Nevelos J.E., Ingham E., Doyle C., Nevelos A.B., Fisher J. (2001). The influence of acetabular cup angle on the wear of “BIOLOX Forte” alumina ceramic bearing couples in a hip joint simulator. J Mater Sci Mater Med.

[14] Firkins P.J., Tipper J.L., Saadatzadeh M.R. (2001). Quantitative analysis of wear and wear debris from metal-on-metal hip prostheses tested in a physiological hip joint simulator. Biomed Mater Eng.

[15] Hood R.W., Wright T.M., Burstein A.H. (1983). Retrieval analysis of total knee prostheses: a method and its application to 48 total condylar prostheses. J Biomed Mater Res.

[16] Fisher J., Reeves E.A., Isaac G.H., Saum K.A., Sanford W.M. (1997). Comparison of the wear of aged and non-aged ultrahigh molecular weight polyethylene sterilized by gamma irradiation and by gas plasma. J Mater Sci Mater Med.

[17] Nevelos J.E., Ingham E., Doyle C., Nevelos A.B., Fisher J. (1999). Analysis of retrieved alumina ceramic components for Mittelmeier total hip prostheses. Biomaterials.

[18] Nevelos J.E., Prudhommeaux Hamadouche M., Doyle C. (2001). Comparative analysis of two different types of alumina-alumina hip prosthesis retrieved for aseptic loosening. J Bone Joint Surg.

[19] Stewart T., Tipper J.L., Streicher R., Ingham E., Fisher J. (2001). Long-term wear or HIPed alumina on alumina bearings for THR under microseparation conditions. J Mater Sci Mater Med.

[20] Hatton A., Nevelos J.E., Nevelos A.A., Banks R.E., Fisher J., Ingham E. (2002). Alumina-alumina artificial hip joints. Part I: a histological analysis and characterisation of wear debris by laser capture micro-dissection of tissues retrieved at revision. Biomaterials.

[21] Williams S., Butterfield M., Stewart T., Ingham E., Stone M.H., Fisher J. (2003). Wear and deformation of ceramic-on-polyethylene total hip replacements with joint laxity and swing phase microseparation. Proc Inst Mech Eng.

[22] Williams S., Stewart T.D., Ingham E., Stone M.H., Fisher J. (2004). Metal-on-metal bearing wear with different swing phase loads. Biomed Mater Res Part B.

[23] Fisher J., Jin Z., Tipper J., Stone M., Ingham E. (2006). Tribology of alternative bearings. Clin Orthop Relat Res.

[24] Williams S., Leslie I., Isaac G., Jin Z., Ingham E., Fisher J. (2008). Tribology and wear of metal-on-metal hip prostheses: influence of cup angle and head position. J Bone Joint Surg A.

[25] Leslie I.J., Williams S., Isaac G., Ingham E., Fisher J. (2009). High cup angle and microseparation increase the wear of hip surface replacements. Clin Orthop Relat Res.

[26] Al-Hajjar M., Leslie I.J., Tipper J., Williams S., Fisher J., Jennings L.M. (2010). Effect of cup inclination angle during microseparation and rim loading on the wear of BIOLOX^®^ delta ceramic-on-ceramic total hip replacement. J Biomed Mater Res Part B Appl Biomater.

[27] Fisher J. (2011). Bioengineering reasons for failure of metal on metal hip prostheses, a bioengineers perspective. J Bone Joint Surg.

[28] Firkins P.J., Tipper J.L., Ingham E., Stone M.H., Farrar R., Fisher J. (2001). Influence of simulator kinematics on the wear of metal-on-metal hip prostheses. Proc Inst Mech Eng.

[29] Brockett C., Williams S., Jin Z.M., Isaac G., Fisher J. (2007). Friction of total hip replacements with different bearings and loading conditions. J Biomed Mater Res Part B Appl Biomater.

[30] Williams S., Jalai-Vahid D., Brockett C.L. (2006). Effect of swing phase load on metal-on-metal hip lubrication, friction and wear. J Biomech.

[31] McEwen H.M.J., Barnett P.I., Bell C.J. (2005). The influence of design, materials and kinematics on the in vitro wear of total knee replacements. J Biomech.

[32] Fisher J., Jennings L.M., Galvin A.L., Jin Z.M., Stone M.H., Ingham E. (2010). 2009 Knee Society presidential guest lecture: polyethylene wear in total knees. Clin Orthop Relat Res.

[33] Jennings L.M., Bell C.J., Ingham E., Komistek R.D., Stone M.H., Fisher J. (2007). The influence of femoral condylar lift-off on the wear of artificial knee joints. J Eng Med Proc Inst Mech Eng.

[34] Bell J., Tipper J.L., Ingham E., Stone M.H., Fisher J. (2001). The influence of phospholipid concentration in protein-containing lubricants on the wear of ultra-high molecular weight polyethylene in artificial hip joints. Proc Inst Mech Eng.

[35] Glyn-Jones S., Isaac S., Hauptfleisch J., McLardy-Smith P., Murray D.W., Gill H.S. (2008). Does highly cross-linked polyethylene wear less than conventional polyethylene in total hip arthroplasty?. J Arthroplasty.

[36] Williams S., Schepers A., Isaac G. (2007). The 2007 Otto Aufranc Award. Ceramic-on-metal hip arthroplasties: a comparative in vitro and in vivo study. Clin Orthop Relat Res.

[37] Leslie I., Williams S., Brown C. (2008). Effect of bearing size on the long-term wear, wear debris, and ion levels of large diameter metal-on-metal hip replacements – an in vitro study. J Biomed Mater Res Part B Appl Biomater.

[38] Manning D.W., Chiang P.P., Martell J.M., Galante J.O., Harris W.H. (2005). In vivo comparative wear study of traditional and highly cross-linked polyethylene in total hip arthroplasty. J Arthroplasty.

[39] Bashyal R.K., Eberhardt J., Malchau H. (2011). Highly crosslinked ultrahigh molecular weight polyethylene in total hip arthroplasty: no further concerns-affirms. Semin Arthroplasty.

[40] Premnath V., Harris W.H., Jasty M., Merrill E.W. (1996). Gamma sterilization of UHMWPE articular implants: an analysis of the oxidation problem. Biomaterials.

[41] Kurtz S.M., Muratoglu O.K., Evans M., Edidin A.A. (1999). Advances in the processing, sterilization, and crosslinking of ultra-high molecular weight polyethylene for total joint arthroplasty. Biomaterials.

[42] Leslie I. Surface engineered hip prostheses. PhD thesis. University of Leeds, 2008.

[43] Minakawa H., Stone M.H., Wroblewski B.M., Lancaster J.G., Ingham E., Fisher J. (1998). Quantification of third-body damage and its effect on UHMWPE wear with different types of femoral head. J Bone Joint Surg.

[44] Galvin A.L., Jennings L.M., Tipper J.L., Ingham E., Fisher J. (2010). Wear and creep of highly cross linked polyethylene against cobalt chrome and ceramic femoral heads. Proc Inst Mech Eng.

[45] Nevelos J., Ingham E., Doyle C. (2000). Microseparation of the centers of alumina-alumna artificial hip joints during simulator testing produces clinically relevant wear and patterns. J Arthroplasty.

[46] Stiehl J.B., Komistek R.D., Dennis D.A., Paxson R.D., Hoff W.A. (1995). Fluoroscopic analysis of kinematics after posterior–cruciate retaining knee arthroplasty. J Bone Joint Surg.

